# Increased Pathologic Downstaging with Induction versus Consolidation Chemotherapy in Patients with Locally Advanced Rectal Cancer Treated with Total Neoadjuvant Therapy—A National Cancer Database Analysis

**DOI:** 10.3390/jcm13030781

**Published:** 2024-01-29

**Authors:** Austin Fan, Beiqun Zhao, Peter Vu, Benjamin Abbadessa, Nicole Lopez, Samuel Eisenstein, Sonia Ramamoorthy, Shanglei Liu

**Affiliations:** 1 Department of Surgery, Division of Colon and Rectal Surgery, School of Medicine, University of California, San Diego, CA 92093, USA; aufan@health.ucsd.edu; 2 Department of Surgery, Division of Colon and Rectal Surgery, University of California, San Diego, CA 92093, USA

**Keywords:** rectal cancer, total neoadjuvant therapy, National Cancer Database

## Abstract

Total neoadjuvant therapy (TNT) is the recommended treatment for locally advanced rectal cancer. The optimal sequence of TNT is debated: induction (chemotherapy first) or consolidation (chemoradiation first)? We aim to evaluate the practice patterns and clinical outcomes of total neoadjuvant therapy with either induction or consolidation regiments in the United States for patients with locally advanced rectal cancer. Methods: This is a retrospective analysis of the National Cancer Database for patients with clinical stage II or stage III rectal cancer, diagnosed between 2006 and 2017, who underwent total neoadjuvant therapy followed by surgery. Results: From 2006 to 2017, we identified 8999 patients and found that the utilization of induction chemotherapy increased from 2.0% to 35.0%. TNT resulted in pathologic downstaging 46.7% of the time and a pathologic complete response 11.6% of the time. Induction chemotherapy lead to higher pathologic downstaging (58% vs. 44.7%, *p* < 0.001) and pathologic complete responses (16.8% vs. 10.7%, *p* < 0.001). Similar trends held true in a multivariate analysis and subset analysis of stage II and III disease. Conclusions: These findings suggest that induction chemotherapy may be preferred over consolidation chemotherapy when downstaging prior to oncologic resection is desired. The optimal treatment plan for total neoadjuvant therapy is multi-factorial and requires further elucidation.

## 1. Introduction

Colorectal cancer (CRC) is the third most prevalent cancer (10.0%) and the second-greatest cause of cancer-related deaths worldwide (9.8%) [1,2]. From 2008 to 2017, the colorectal cancer death rate rose 1.3% per year for adults under the age of 55 [2]. The determination of an optimal treatment plan for an individual patient is a comprehensive decision weighing various factors, including rates of fecal incontinence, minimizing cancer recurrence, restoring normal bowel function, sexual dysfunction, and protecting genitourinary function [3]. With regard to locally advanced rectal cancer (LARC), particularly stage II (T3-4, node-negative disease) or stage III (node-positive disease without distant metastasis) rectal cancer, a combination of chemoradiotherapy (capecitabine or 5-fluorouracil with radiation therapy) and systemic chemotherapy (such as 5-fluorouracil, leucovorin, and oxaliplatin with or without irinotecan) can be utilized due to the relatively high risk of locoregional recurrence [3].

Historically, the treatment for LARC consisted of neoadjuvant chemoradiotherapy (nCRT) followed by restaging and possible surgical resection, and then, if necessary, adjuvant systemic chemotherapy [3,4]. In the past two decades, nCRT plus systemic chemotherapy prior to surgery, also referred to as total neoadjuvant therapy (TNT), has become a popular treatment strategy for LARC and has become the recommended treatment modality by the National Comprehensive Cancer Network (NCCN) [5,6,7,8]. When compared to the traditional algorithm, TNT offers multiple advantages, such as improving the tolerance and completion rates of chemotherapy, greater T and N downstaging, early prevention or the elimination of micrometastases, an increased R0 resection rate, and a higher proportion of pathologic complete responses (pCRs) [3,5,6,7,8]. 

However, the order of chemoradiotherapy and systemic chemotherapy in TNT is still widely debated. There are two TNT sequences: induction chemotherapy (IC), which involves systemic chemotherapy before chemoradiation, and consolidation chemotherapy (CC), which involves systemic chemotherapy after chemoradiation [5]. While both the induction chemotherapy and consolidation chemotherapy schedules have reported benefits, the optimal sequence of IC and CC is still under active discussion and has not been well defined [5,9,10,11]. 

Although randomized trials remain the benchmark standard of evidence, they are not without limitations, including strict selection criteria, adherence to rigorous follow-up schedules, and restriction to highly specialized surgeons and care centers. Retrospective observation studies provide an important addition to ensure that results from randomized trials are reflective of outcomes in practice. We performed a retrospective cohort study using the National Cancer Database (NCDB). The data were sourced from over 1500 Commission-on-Cancer (CoC)-accredited healthcare centers and includes approximately 70% of all newly diagnosed cancer cases in the United States [12]. Our study evaluates practice changes and outcomes in patients with locally advanced rectal cancer (LARC) undergoing either induction chemotherapy (IC) or consolidation chemotherapy (CC) when treated with TNT. 

## 2. Materials and Methods

### 2.1. Data

A retrospective cohort study was performed using the National Cancer Database (NCDB). The NCDB, jointly sponsored by the American College of Surgeons and the American Cancer Society, is a national outcomes database that is focused on clinical oncologic data. Institutional Review Board exemption was granted for this study due to the de-identified nature of the data. 

### 2.2. Cohort

Patients diagnosed with rectal adenocarcinoma were identified using International Classification of Disease for Oncology histology codes. All patients diagnosed with clinical stage II or III rectal adenocarcinoma between 2006 and 2017 were included in this analysis. Patients with metastatic disease and unknown stages were excluded. Unfortunately, the NCDB does not include surveillance imaging data, nor does it delineate the outcomes of patients undergoing a watch-and-wait follow-up protocol. As a result, patients who did not undergo surgical excision were excluded from the pGD and pCR analysis. 

**Figure 1:**
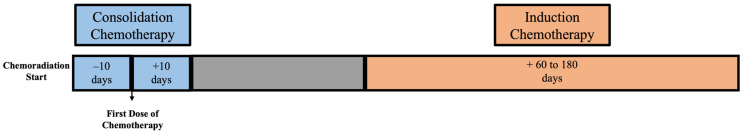
Timeline defining consolidation and induction chemotherapy. Consolidation chemotherapy was defined as starting chemotherapy and radiation within 10 days of each other. Induction therapy was defined as radiation starting between 60 and 180 days after chemotherapy.

All patients in our cohort received TNT. The cohort was split into two groups: IC versus CC. The IC group included patients treated with multiagent chemotherapy prior to chemoradiation, while the CC group included patients treated with chemoradiation prior to multiagent chemotherapy. Patients receiving radiotherapy without a concurrent radiosensitizing chemotherapy were excluded from our study, as this did not qualify as TNT chemoradiation. Similarly to previously published studies [13], the groups were identified by comparing the days from diagnosis to chemotherapy and/or to radiation. CC was defined as starting chemotherapy and radiation within 10 days of each other because this symbolizes an intention to start radiation at the beginning of TNT. If radiation was started between 60 and 180 days after chemotherapy, this was defined as IC, as this symbolizes a minimum of 8 weeks of intentional chemotherapy prior to the initiation of radiation. Although the NCDB does not provide information on the number of chemotherapy cycles, our primary focus was concentrated on the intention to treat. Patients who underwent radiation >10 days before chemotherapy or >180 days after chemotherapy were excluded from this analysis, as these represented significant TNT protocol deviation (Figure 1). 

### 2.3. Variables

Patient demographic variables included age, sex, and race. The patient’s age was dichotomized to less than 65 and greater than 65 years old. Race was categorized into White, Black, Asian/Pacific Islander, and other/unknown (which includes Native Americans). Clinical variables included the Charlson–Deyo score, clinical T stage, clinical N stage, and tumor grade. The Charlson–Deyo score, a representation of patient comorbidities, is categorized into 0, 1, 2, or 3. The tumor grade is categorized into well-differentiated, moderately differentiated, poorly differentiated, or unknown. The year of diagnosis was evenly split into three groups over the 12-year period. Lastly, the treatment facility was also included. In the NCDB, the treatment facility type is categorized into academic comprehensive cancer programs, community cancer programs (100–500 newly diagnosed cancer cases a year), comprehensive community cancer programs (greater than 500 newly diagnosed cancer cases a year), and integrated network cancer programs.

#### Statistical Analysis

The primary goal of our study was to compare IC versus CC in terms of (1) pathologic group downstaging (pGD), as defined by a decrease in the clinical to pathologic stage group; and (2) pathologic complete response (pCR), as defined by pT0N0. Secondary outcomes included overall survival (OS), the tumor margin, and the circumferential resection margin (CRM). A univariable analysis was performed using the chi-square test. A survival analysis was performed using a Kaplan–Meier analysis. A multivariable logistic regression with backward stepwise selection (threshold of *p* < 0.20, determined a priori) was performed to identify independent predictors of pathologic downstaging and pCR. Sub-analyses of clinical stage II and stage III patients were also performed. All analyses were performed using IBM SPSS Statistics (IBM Corporation, Version 26, Armonk, NY, USA). All statistical tests were 2-sided, and the level of significance was set at 0.05 for all analyses.

## 3. Results

**Figure 2:**
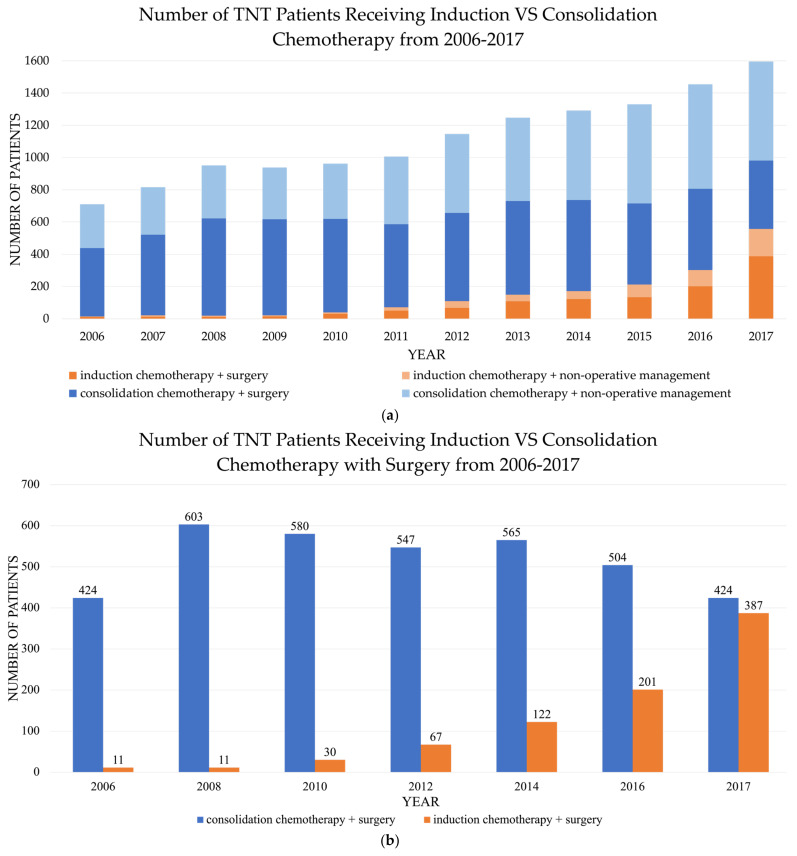
(**a**) Number of total neoadjuvant therapy (TNT) patients reported in the NCDB receiving induction chemotherapy vs. consolidation chemotherapy from 2006 to 2017. (**b**) Number of total neoadjuvant therapy (TNT) patients reported in the NCDB receiving induction chemotherapy vs. consolidation chemotherapy with surgery from 2006 to 2017.

We identified 16,998 patients with locally advanced rectal cancer (LARC) who underwent TNT, of which 8889 had surgical resection (Table 1). In this cohort, 1146 (12.9%) patients received IC and 6342 (71.3%) patients received CC. A total of 1401 (15.8%) patients were excluded from the analysis due to deviation from standard TNT protocol. The percentage of total patients receiving IC increased from 2.0% in 2006 to 35.0% in 2017, with a corresponding drop in the percentage of patients receiving CC (Figure 2). When accounting only for patients who underwent definitive surgery after TNT, the number receiving IC increased even more from 2.5% in 2006 to 47.7% in 2017.

For patients undergoing surgery, 2568 (46.7%) patients had experienced pGD and 868 (11.6%) had pCR at final pathology after TNT regardless of the order of treatment. The final surgical pathology and overall survival for IC vs. CC in LARC and a breakdown by stage can be seen in Table 2. For both stage II and III disease, IC patients experienced a higher rate of pGD (58% vs. 44.7%, *p* < 0.001) and pCR (16.8% vs. 10.7%, *p* < 0.001). The same trend was seen in the subset analysis of stage III disease for pGD (62.6% vs. 53.0%, *p* < 0.001) and pCR (16.5% vs. 11.0%, *p* < 0.001). However, in stage II disease, only pCR was significantly improved in the IC treatment group (17.3% vs. 10.2%, *p* < 0.001). Similarly, there was a higher chance of downstaging the tumor’s T stage in the IC group compared to CC group across all analysis. Regarding the tumor margin status, there was a significantly higher rate of positive CRM in the IC group in the combined stage II and III group (6.3% vs. 4.2%, *p* = 0.01). This trend was also observed in a separate analysis of stage III patients (6.5% vs. 4.4%, *p* = 0.028). There was no difference in overall survival.

To confirm the effect of IC vs. CC on pGD and pCR, a multivariable logistic regression analysis was performed accounting for the tumor stage, grade, timing of diagnosis, treatment facility, and various baseline demographics (Table 3). IC was still significantly associated with pGD compared to CC, with an adjusted odds ratio of 1.390 (*p* < 0.001, 95% CI 1.176–1.643), and similarly for pCR, with an adjusted odds ratio of 1.220 (*p* = 0.047, 95% CI 1.003–1.484). Interestingly, patients treated in the later one-third of our analysis (2014–2017) also had improved pGD (aOR 1.186, *p* = 0.023, 95% CI 1.023–1.375) and pCR (aOR 3.269, *p* < 0.001, 95% CI 2.608–4.098). The analysis of treatment facilities also showed decreased pCR rates at community centers (aOR 0.533, *p* < 0.001, 95% CI 0.379–0.749) and comprehensive community centers (aOR 0.745, *p* < 0.001, 95% CI 0.626–0.886) compared to academic centers. However, the same trend was only significant for community centers in terms of pGD (aOR 0.786, *p* = 0.038, 95% CI 0.625–0.987).

## 4. Discussion

Although there is largely agreement in terms of the importance and benefit of TNT for the treatment of LARC, the order of chemotherapy versus chemoradiation is still undergoing evaluation. To our knowledge, this is the first large, national database study to evaluate the effect of IC versus CC. In addition to evaluating the clinical outcomes, we also seek to describe the changing practice pattern over the last decade. 

We identified a significant increase in the prevalence of IC, going from 2% in 2006 to 35% in 2017, while CC had only modest increases. The largest increase in IC was seen after 2014, at a time when the NCCN was beginning to adopt TNT for its treatment recommendation for LARC with recognition that IC may be beneficial in cases of micrometastasis [3]. The recent distribution of IC and CC in 2017 indicate that both TNT schedules are being similarly utilized, and that an optimal schedule has still not been determined [3,5]. Furthermore, although we cannot definitively conclude whether CC or IC is more preferred in the clinical setting, it is evident that CC is still more widely practiced despite the fact that IC has gained popularity in our study period. It is difficult to pinpoint a specific reason for the increased prevalence of IC, but we speculate that it may be due to a multitude of reasons. First, previous large clinical trials (e.g., PRODIGE23, GCR-3) that were investigating TNT utilized IC as their primary treatment plan [14,15]. This likely influenced the treatment protocols in many institutions during the earlier years of TNT, as their protocols were likely established to emulate these trials. It is also possible that outside of highly controlled clinical trials, IC may be logistically preferred due to the local availability of medical oncology versus radiation oncology, affecting referral patterns after a diagnosis of LARC. 

In our analysis, we showed that, in the univariate analysis, stage II/stage III LARC showed increased rates of pGD, pCR, and T stage downgrade for IC compared to CC in TNT patients who underwent surgical resection. The multivariable logistic regression analysis reinforced the finding that IC was associated with significantly higher pGD and pCR for LARC after accounting for confounders of clinical staging, patient demographics, and the classification of treatment facilities. One possible reason for this is the higher adherence to IC strategies due to the shorter total time of radiation-related toxicity. A previous study has shown higher rates of neoadjuvant chemotherapy completion when administered prior to radiation [16]. Furthermore, the results from the surgical outcomes of the RAPIDO trial indicated that CC was associated with higher perioperative blood loss and lower rates of an intact mesorectal plane when compared to surgery following chemoradiation without significant delay [17,18]. These differences in outcomes were attributed to a longer delay between radiotherapy and surgery in CC, ultimately leading to more difficult surgical resection. Although this was not a study comparing the surgical complications between IC and CC, IC would confer the same benefits of a shorter time from radiation to surgery. Additionally, it is valuable to emphasize that our patient population excludes patients who did not undergo surgical excision and underwent a watch-and-wait follow-up protocol. Patients undergoing a watch-and-wait follow-up protocol achieved a clinical complete response (cCR) after chemoradiotherapy [19]. Therefore, our findings are primarily applicable to individuals that are not expected to have a clinical complete response (cCR) due to bulky or advanced disease. 

Surprisingly, our data also suggested in the univariate analysis that IC was associated with a higher rate of CRM positivity compared to CC in the combined LARC cohort. One may postulate that this is due to the benefit of a prolonged wait period after radiation to allow further tumor shrinkage, as suggested in prior studies [15,18]. However, the occurrence of a positive CRM after surgery tends to be low (between 4.2 and 6.3%) and is more likely to be affected by surgical technique compared to pCR and pGD. Thus, it is difficult to comment on the strength of this observation. Still, future research is warranted to be better powered to answer this question. Ultimately, as consistent with previous research, there was no observed difference in the mean OS [20].

Overall, these findings suggest that if pathologic downstaging is desired prior to oncologic resection, IC may be preferred over CC. However, if the patient presents with any indication of threatened circumferential margins, CC may be preferred over IC. This is a surprising finding given recent randomized controlled trials from Europe describing differing results: higher pCR rates for CC compared to IC [16]. The CAO/ARO/AIO-12 reported a higher pCR rate for CC compared to IC (25% vs. 17%), suspecting that the prolonged interval between the completion of CRT and surgery may have contributed to their observed higher pCR [16]. Furthermore, although the OPRA trial did indicate a significantly higher cCR rate with CC compared to IC (53% vs. 41%, *p* = 0.01), their study did not indicate any significant differences in pCR between IC and CC [20]. Our analysis of the NCDB seems to provide an alternative conclusion to results from these previous works. We suspect this is due to a multitude of factors. First is the translatability of the randomized controlled trial (RCT) population to general practice. Due to a strict selection criteria and the commitment of the population in RCTs to adhere to rigorous follow-up schedules, there may exist confounding factors in the care of cancer patients in the community that is not accurately reflected in a trial. A previous study reported that rectal cancer patients experienced a median diagnostic delay of 43 days and a median treatment delay of 18 days [19]. On the other hand, RCTs such as RAPIDO reported a median time of 14 days between the conclusion of radiotherapy and the start of chemotherapy [18]. Second, treatment adherence also plays a major role in the success of TNT. IC may be better tolerated than CC, as noted by NCCN guidelines and by the CAO/ARO/AIO-12 trial [3,15]. It may be more difficult for a patient to adhere to CC outside of an RCT. Although RCTs remain the gold standard, retrospective studies are necessary to ensure that the results from RCTs are reflective of outcomes in broad clinical practice in the community. The NCDB data represent the effect of treatment compliance on practices across the United States, ultimately highlighting the importance of complex decision making for treatment order. Logistic barriers to care and complications to therapy should be taken in consideration in the decision of TNT. We believe that having a tailored approach to IC versus CC depending on the expected treatment course can lead to the best outcomes.

### Limitations

Our study is limited by its retrospective nature, which can limit the variables available for analysis. For rectal cancer, the NCDB does not include variables for disease-free survival, local recurrence, methods for clinical staging (e.g., MRI, CT, ERUS), or specific chemotherapy regimens and durations (e.g., the number of cycles of chemotherapy). In addition, we are unable to explicitly determine the specific neoadjuvant radiation regimen (e.g., long-course versus short-course), the tumor location within the rectum, treatment-related complications, specific reasons for TNT deviation, or occurrences of metastatic disease during treatment.

## 5. Conclusions

Using a US nationwide cohort, our study reports that the utilization of induction chemotherapy in total neoadjuvant therapy has had a faster adoption compared to consolidation chemotherapy in recent years. Our study suggests that, when compared to consolidation, induction chemotherapy is associated with improved pathologic downstaging and pathologic complete response in patients with locally advanced rectal cancer treated with total neoadjuvant therapy prior to surgical resection. Our study suggests that induction chemotherapy may be preferred over consolidation chemotherapy when pathologic downstaging is desired prior to oncologic resection, especially when the circumferential surgical margins are not threatened. However, further studies are necessary to understand the effect of compliance on decision making and clinical outcomes. 

## Figures and Tables

**Table 1:** Patient demographics.

Variable	Induction (%)	Consolidation (%)
Age ≥65	237 (20.7)	1997 (31.5)
Female Sex	424 (37.0)	2370 (37.4)
Race		
White	955 (83.3)	5519 (87.0)
Black	86 (7.5)	510 (8.0)
Asian/Pacific Islander	60 (5.2)	201 (3.2)
Other/Unknown	45 (3.9)	112 (1.8)
Charlson-Deyo		
0	961 (83.9)	5066 (79.9)
1	154 (13.4)	994 (15.7)
2	23 (2.0)	209 (3.3)
≥3	8 (0.7)	73 (1.2)
Clinical T Stage		
cT0/1	5 (0.3)	61 (1.0)
cT2	56 (4.9)	370 (5.8)
cT3	823 (71.8)	5246 (82.7)
cT4	254 (22.2)	572 (9.0)
cTx	9 (0.8)	93 (1.5)
Clinical N Stage		
cN0	219 (19.1)	2674 (42.2)
cN1	592 (51.7)	2892 (45.6)
cN2	328 (28.6)	638 (10.1)
cNx	7 (0.6)	138 (2.2)
Tumor Grade		
Well-differentiated	57 (5.0)	527 (8.3)
Moderately-differentiated	733 (67.5)	4212 (66.4)
Poorly-differentiated	111 (9.7)	708 (11.2)
Grade Unknown	205 (17.9)	895 (14.1)
Year of Diagnosis		
2006–2009	48 (4.2)	2123 (33.5)
2010–2013	255 (22.3)	2223 (35.1)
2014–2017	843 (73.6)	1996 (31.5)
Treatment Facility		
Academic	637 (55.6)	1954 (30.8)
Community	38 (3.3)	517 (8.2)
Comprehensive Community	186 (16.2)	2585 (40.8)
Integrated Network	181 (15.8)	920 (14.5)
Unknown	104 (9.1)	366 (5.8)

**Table 2:** Pathologic outcomes and overall survival in LARC between IC and CC.

	Induction Chemotherapy (%)	Consolidation Chemotherapy (%)	*p*-Value	OR (95% CI)
Stage II/III				
pGD	495 (58.0)	2073 (44.7)	**<0.001**	1.709 (1.474–1.981)
pCR	192 (16.8)	676 (10.7)	**<0.001**	1.687 (1.417–2.008)
Mural Margin (+)	62 (5.5)	375 (6.1)	0.454	-
CRM (+)	53 (6.3)	233 (4.2)	**0.01**	1.519 (1.117–2.065)
Decreasing T Stage	610 (62.8)	2454 (51.4)	**<0.001**	1.593 (1.382–1.836)
Mean OS	107.2 months	114.5 months	0.062	-
Stage II Only				
pGD	56 (36.6)	652 (33.2)	0.423	-
pCR	39 (17.9)	279 (10.2)	**<0.001**	1.923 (1.331–2.779)
Mural Margin (+)	6 (2.8)	140 (5.2)	0.143	-
CRM (+)	9 (5.3)	95 (3.9)	0.416	-
Decreasing T Stage	126 (68.1)	1098 (54.0)	**<0.001**	1.822 (1.322–2.513)
Stage III Only				
pGD	439 (62.6)	1421 (53.0)	**<0.001**	1.483 (1.250–1.760)
pCR	153 (16.5)	397 (11.0)	**<0.001**	1.593 (1.301–1.950)
Mural Margin (+)	56 (6.1)	235 (6.7)	0.53	-
CRM (+)	44 (6.5)	138 (4.4)	**0.028**	1.503 (1.059–2.133)
Decreasing T Stage	484 (61.5)	1356 (49.5)	**<0.001**	1.629 (1.386–1.916)
Decreasing N Stage	676 (78.1)	1914 (64.5)	**<0.001**	1.957 (1.639–2.338)

OR, odds ratio; CI, confidence interval; pGD, pathological downstaging; pCR, pathologic complete response; CRM, circumferential resection margin; OS, overall survival. Bold values are statistically significant.

**Table 3:** Multivariable regression of pGD and pCR.

	pGD		pCR	
Variables	*p*-Value	aOR (95% CI)	*p*-Value	aOR (95% CI)
Female Sex	0.175	-	-	-
Race				
White	-	-	Ref.	
Black	-	-	0.035	0.730 (0.545–0.978)
Asian/Pacific Islander	-	-	0.345	-
Other/Unknown	-	-	0.523	-
Charlson-Deyo Score				
0	Ref.		-	-
1	0.491	-	-	-
2	0.024	1.439 (1.048–1.976)	-	-
≥3	0.152	-	-	-
Clinical T Stage				
cT0/1	Ref.		Ref.	
cT2	0.011	2.287 (1.211–4.319)	0.246	-
cT3	0.004	2.369 (1.307–4.294)	0.714	-
cT4	0.075	-	0.195	-
cTx	0.013	2.766 (1.242–6.161)	0.482	-
Clinical N Stage				
cN0	Ref.		Ref.	
cN1	<0.001	2.487 (2.194–2.818)	0.766	-
cN2	<0.001	1.875 (1.561–2.251)	0.457	-
cNx	0.334	-	0.03	0.203 (0.048–0.858)
Tumor Grade				
Well-differentiated	Ref.		Ref.	
Moderately-differentiated	0.339	-	0.641	-
Poorly-differentiated	<0.001	0.402 (0.309–0.523)	0.163	-
Grade Unknown	0.886	-	0.002	1.630 (1.197–2.220)
Year of Diagnosis				
2006–2009	Ref.		Ref.	
2010–2013	0.092	-	<0.001	2.477 (1.972–3.111)
2014–2017	0.023	1.186 (1.023–1.375)	<0.001	3.269 (2.608–4.098)
Treatment Facility				
Academic	Ref.		Ref.	
Community	0.038	0.786 (0.625–0.987)	<0.001	0.533 (0.379–0.749)
Comprehensive Community	0.071	-	<0.001	0.745 (0.626–0.886)
Integrated Network	0.256	-	0.09	-
Unknown	0.01	0.728 (0.572–0.926)	0.089	-
Induction Chemotherapy	**<0.001**	1.390 (1.176–1.643)	**0.047**	1.220 (1.003–1.484)
Removed by Stepwise Selection	Age ≥ 65, Race		Age ≥ 65, Female Sex, Charlson-Deyo Score	

aOR, adjusted odds ratio; CI, confidence interval; pGD, pathological downstaging; pCR, pathologic complete response. Bold values are statistically significant.

## Data Availability

Restrictions apply to the availability of these data. Data was obtained from American College of Surgeons and are available at https://www.facs.org/quality-programs/cancer-programs/national-cancer-database/ with the permission of the American College of Surgeons.

## Abbreviations

aOR	adjusted odds ratio
CRM	circumferential resection margin
cCR	clinical complete response
CRC	colorectal cancer
CoC	Commission on Cancer
CT	computed tomography
CI	confidence interval
CC	consolidation chemotherapy
ERUS	endorectal ultrasonography
IC	induction chemotherapy
LARC	locally advanced rectal cancer
MRI	magnetic resonance imaging
NCDB	National Cancer Database
NCCN	National Comprehensive Cancer Network
nCRT	neoadjuvant chemoradiotherapy
NOM	non-operative management
OR	odds ratio
OS	overall survival
pCR	pathologic complete response
pGD	pathologic group downstaging
RCT	randomized controlled trial
TNT	total neoadjuvant therapy
